# Gut microbiota–innate immune crosstalk in the initiation and progression of CRC: mechanisms and therapeutic potential

**DOI:** 10.3389/fimmu.2026.1892843

**Published:** 2026-09-09

**Authors:** Jingjie Lin, Huirong Lin, Zhang Zhang, Hengmin Wang

**Affiliations:** 1 Department of Gastrointestinal Surgery, The First People’s Hospital of Wenling, Affiliated Wenling Hospital, Wenzhou Medical University, Wenling, Zhejiang, China; 2 Department of Traditional Chinese Pharmacy, Taizhou Cancer Hospital, Taizhou, Zhejiang, China; 3 Department of Gastroenterology, Shulan (Hangzhou) Hospital, Shulan International Medical College, Zhejiang Shuren University, Hangzhou, China

**Keywords:** CRC, gut microbiota, innate immunity, microbiota-based therapy, mucosal barrier, tumor immune microenvironment

## Abstract

Colorectal cancer (CRC) is a common malignancy of the digestive system, and its initiation and progression are associated with multiple factors, including genetic alterations, environmental exposures, changes in the gut microbiota, and disruption of immune homeostasis. In recent years, evidence from human observational studies, animal models, and *in vitro* experiments has shown that the gut microbiota can influence Toll-like receptor and NOD-like receptor signaling, NF-κB activation, the NLRP3 inflammasome, and immunometabolic pathways through multiple mechanisms, including microbial structural components, metabolic signals, virulence factors, and outer membrane vesicles. These effects are associated with functional remodeling of innate immune components such as macrophages, dendritic cells, neutrophils, and natural killer (NK) cells. Conversely, the innate immune system can reshape microbial composition, spatial distribution, and metabolic characteristics through antimicrobial effector molecules, maintenance of the mucosal barrier, release of inflammatory mediators, and phagocytic clearance, indicating a bidirectional regulatory relationship between the gut microbiota and innate immunity. Current evidence suggests that microbiota–innate immune interactions are associated, to varying degrees, with CRC-related pathological processes, including alterations in immune surveillance and the myeloid microenvironment, barrier dysfunction, low-grade inflammation, remodeling of the tumor microenvironment, epithelial–mesenchymal transition-related phenotypes, and immune evasion. Notably, colibactin-associated mutational signatures have been directly demonstrated in the genomes of human CRC tumors, representing one of the relatively rare forms of high-strength mechanistic evidence in microbiota–CRC research. Beyond such a limited number of well-supported mechanisms, however, many specific microbiota–immune interactions and their roles at different pathological stages remain based primarily on animal models, *in vitro* experiments, or integrative inference, while longitudinal causal evidence in humans remains generally limited. This review focuses on the bidirectional interactions between the gut microbiota and innate immunity in CRC, systematically summarizing their molecular mechanisms, associated pathological processes, and advances in microbiota-targeted interventions, with particular emphasis on distinguishing among different types of evidence, experimental contexts, and levels of causal certainty. In doing so, we aim to provide a more cautious theoretical basis for mechanistic research on the microbiota–immune axis and for the development of precision intervention strategies in CRC.

## 1 Introduction

Colorectal cancer (CRC) is one of the most common malignancies of the digestive system (1). Its initiation and progression involve complex pathological processes, including aberrant proliferation of the intestinal epithelium, adenoma-to-carcinoma transformation, local invasion, and distant metastasis, and are closely associated with intestinal dysfunction, tumor recurrence, and patient survival outcomes (2–4). Traditionally, CRC has been considered to be associated with factors such as the accumulation of genetic mutations, unhealthy dietary patterns, chronic inflammatory stimulation, and environmental exposures (5, 6). However, these factors alone are insufficient to fully explain the marked interindividual differences and tumor heterogeneity observed among patients with CRC. Recent studies have further shown that, in addition to tumor cell-intrinsic genetic and epigenetic abnormalities, CRC is frequently accompanied by disruption of the mucosal barrier, persistent low-grade inflammation, and remodeling of the tumor immune microenvironment (7, 8). Accordingly, alterations in the gut microbial ecosystem and dysregulated immune responses are considered to potentially contribute to CRC-related pathological processes.

CRC also exhibits substantial molecular and immune heterogeneity. Within the consensus molecular subtype (CMS) classification, CMS1, also referred to as the MSI–immune subtype, is characterized by a high mutational burden, microsatellite instability, and prominent immune activation. Distinct immune microenvironmental features are also observed between MSI-H/dMMR tumors and MSS/pMMR tumors, the latter accounting for the majority of CRC cases (9). In addition, human studies have shown that a higher abundance of Fusobacterium nucleatum in tumor tissues is associated with MSI-H status and with patterns of local T-cell infiltration (10). These differences suggest that microbiota-related immune effects may be influenced by the molecular subtype and immune context of CRC, further supporting the need to investigate these effects from the perspective of innate immunity, including pattern recognition, myeloid cell function, and the mucosal barrier.

Microbiota-derived signals, including microbial-associated molecules such as lipopolysaccharide and flagellin, as well as metabolites such as short-chain fatty acids, can influence the functional state of innate immunity through pattern-recognition receptor sensing and metabolism-related regulatory mechanisms, thereby contributing to the maintenance of mucosal immune homeostasis (11, 12). Conversely, the innate immune system can influence the composition, spatial distribution, and metabolic activity of the gut microbiota through mechanisms including the secretion of antimicrobial peptides and mucus, the release of inflammatory mediators, and phagocyte-mediated clearance (13, 14). Under CRC-associated conditions, these regulatory processes may be altered and may interact with changes in microbial composition and function, suggesting a dynamic relationship in which microbial dysbiosis and innate immune abnormalities may influence one another (15).

Previous reviews have systematically discussed the gut microbiota and immune regulation in CRC from different perspectives. Liu et al. focused on the bidirectional regulation of the microbiota–immune axis, remodeling of the tumor immune microenvironment, and microbiota-targeted intervention strategies in CRC (16). Xing et al. examined interactions between the gut microbiota and both innate and adaptive immunity from a broader host immune perspective and discussed their implications for inflammatory signaling, tumorigenesis, and immunotherapy (17). Zhang et al. mainly focused on the effects of the gut microbiota on tumor immune surveillance, responses to immunotherapy and chemotherapy, and the potential of the microbiota as a predictive biomarker and therapeutic target in CRC (18). Neagu et al. summarized the effects of CRC-associated microbiota on immune responses, intestinal barrier integrity, and microbiota-based interventions, while also discussing the immune effects of different potentially pathogenic and protective microbial taxa (19). Dong et al. reviewed the relationships among intestinal barrier abnormalities, microbial alterations, chronic inflammation, and related diagnostic and therapeutic strategies within the framework of the barrier–microbiota–inflammation axis (20). Collectively, these reviews have already provided relatively comprehensive coverage of key topics, including microbiota–immune interactions, barrier function, immune surveillance, treatment response, and microbiota-targeted interventions.

Building on this foundation, the present review does not regard bidirectional microbiota–immune interactions themselves as a novel concept, but instead further narrows the scope of analysis to the specific mechanistic links between the gut microbiota and innate immunity. The review is organized around the central framework of microbial signals shaping innate immunity and innate immunity reciprocally remodeling the microbial ecosystem. We summarize the relationships between different microbial signals and pattern recognition, myeloid cell function, and the mucosal barrier, while also discussing how innate immunity reciprocally regulates microbial composition, spatial distribution, and metabolic activity. In addition, by considering strain- or virulence factor-specific effects, the origins of metabolic signals, and different types of evidence, we critically evaluate the scope of applicability and causal boundaries of the relevant mechanisms, thereby providing a focused and structured synthesis of bidirectional microbiota–innate immune interactions in CRC.

This narrative review integrates and analyzes studies investigating gut microbiota–innate immune interactions in colorectal cancer (CRC). Relevant literature was primarily retrieved from databases including PubMed and the Web of Science Core Collection, covering the period from database inception to March 2026. The main search terms included “colorectal cancer,” “gut microbiota,” “microbiome,” “innate immunity,” “pattern recognition receptors,” “microbial metabolites,” “virulence factors,” “extracellular vesicles,” “fungal microbiome,” and “microbiota-targeted interventions,” which were combined according to the specific topic being examined. Priority was given to recent research published within the past five years.

For CRC-related mechanisms and recent advances, we prioritized human studies, animal model studies, *in vitro*/organoid studies, and information from clinical trials published within the past five years. At the same time, because certain classical mechanisms, key microbial virulence factors, major immune signaling pathways, and fundamental metabolic processes could not be adequately covered by studies published within this period alone, representative studies of major mechanistic importance published within the past decade were also included. In addition, high-quality reviews published in recent years were consulted to help establish the research background and integrate evidence across different fields. Studies conducted outside the context of CRC were cited only when they provided important mechanistic support, and their nature as indirect evidence was explicitly indicated in the main text.

## 2 Gut microecological alterations and innate immune homeostatic shifts associated with CRC

In recent years, advances in metagenomics, metabolomics, and spatial omics have enabled increasingly systematic characterization of CRC-associated alterations in the gut microbial ecosystem (21). Compared with healthy individuals, patients with CRC generally exhibit changes in microbial composition and functional potential, although considerable heterogeneity remains across studies (22). Such inconsistencies may be attributable to multiple factors, including dietary patterns, host metabolic status, disease stage, tumor location, sample type, and prior treatment exposure (23). Therefore, taxonomic shifts in microbial abundance alone are insufficient to fully explain the potential roles of the gut microbiota in CRC. Accordingly, current research has increasingly focused on microbial ecological functions, spatial distribution, and adaptive features within the local microenvironment.

Notably, tumor anatomical location represents an important biological context underlying microbial and immune heterogeneity in CRC. Studies of human tumor tissues have shown that proximal and distal CRCs differ in their tumor-associated microbial composition and community structure, with enrichment of Fusobacteria and Fusobacterium nucleatum being more prominent in proximal tumors (24, 25). At the same time, right-sided CRC is more frequently associated with molecular features such as microsatellite instability and BRAF mutations and may exhibit greater local immune cell infiltration (26). Therefore, tumor sidedness should be considered an important biological context when interpreting microbiota–innate immune interactions in CRC, and microbial or immune features observed in specific anatomical sites or patient subgroups should not be directly extrapolated to CRC as a whole.

At the ecological level, CRC-associated microbial alterations are often accompanied by changes in the local intestinal microenvironment. Certain tumor-associated microbial populations may acquire a relative growth advantage within ecological niches shaped by inflammation, hypoxia, altered nutrient availability, and disruption of the mucus layer, thereby becoming enriched in tumor tissues or adjacent mucosa (27, 28). Meanwhile, changes in local oxygen tension, metabolite distribution, and epithelial barrier status may also influence the ecological balance between obligate and facultative anaerobes (29). These niche alterations may not only affect microbial stability and spatial organization but may also increase contact and signaling exchange between microorganisms, epithelial cells, and immune cells, thereby creating a background of sustained microbial–epithelial–immune signal exposure.

At the barrier level, alterations in the microbial ecosystem are associated to some extent with intestinal epithelial barrier dysfunction (30). Reduced expression of tight-junction proteins, changes in mucus layer architecture, and impaired epithelial repair capacity may all be associated with increased intestinal permeability (31). Notably, in some CRC tissues and relevant experimental models, these changes are accompanied by persistent low-grade immune activation and tissue remodeling rather than a typical acute inflammatory response (32). This state may involve complex interactions among tumor cells, stromal cells, epithelial cells, and immune cells, although the mechanisms underlying its development and the direction of causality remain to be further clarified (33).

At the immune level, intestinal innate immune homeostasis may undergo varying degrees of remodeling in CRC-associated states. Under physiological conditions, the mucosal immune system maintains microbial spatial segregation and immune tolerance through multiple mechanisms, including the mucus barrier, antimicrobial molecules, IgA-mediated microbial restriction, and phagocytic clearance (34, 35). Within the CRC tumor microenvironment, immune components such as macrophages, dendritic cells, myeloid-derived suppressor cells, and NK cells may undergo functional alterations and participate in the regulation of local inflammation, immunosuppression, and antitumor immunity (36, 37). Importantly, such immune remodeling may be associated with microbial changes while also exerting reciprocal effects on microbial composition and ecological niches. Within the analytical framework of this review, adaptive immune responses are discussed primarily as downstream effects or amplification steps following the remodeling of innate immunity, myeloid cells, and the mucosal barrier.

Overall, bidirectional associations may exist between alterations in the gut microbiota and the tumor-associated immune microenvironment, while tumor-related inflammation, metabolic reprogramming, and immune selection pressure may also collectively contribute to shaping microbial structure and function. However, the current evidence is still derived mainly from cross-sectional human studies, animal models, and *in vitro* experiments. The temporal sequence, directionality, and causal relationships between microbial alterations and innate immune remodeling therefore remain to be further elucidated.

## 3 Molecular mechanisms and signaling regulation underlying gut microbiota–innate immune crosstalk

The gut microbiota can influence host innate immune recognition and effector responses through diverse microbial-derived components, including structural molecules, metabolic signals, virulence factors, secreted molecules, and extracellular nucleic acids. Conversely, innate immune responses may also shape microbial composition, spatial organization, and metabolic activity through mechanisms involving mucosal barrier regulation, antimicrobial molecules, inflammatory mediators, and phagocytic clearance, thereby establishing a complex bidirectional interaction network. It should be noted that this review primarily focuses on the mechanistic connections between the gut microbiota and innate immune pattern recognition, effector pathways, and myeloid cell functional remodeling. Adaptive immune components, including Tregs, CD8^+^ T cells, immunoglobulin A (IgA), and immune checkpoint responses, are discussed mainly as downstream consequences or amplification processes associated with innate immune regulation, antigen presentation, barrier alterations, and tumor immune microenvironment remodeling, rather than as the central mechanistic focus of this review.

### 3.1 Molecular mechanisms by which the gut microbiota regulates innate immunity

#### 3.1.1 Microbial structural components

Microbial structural components represent an important source of signals through which the host innate immune system recognizes the gut microbiota. These components mainly include lipopolysaccharide (LPS), lipoteichoic acid, peptidoglycan, and flagellin. They can be sensed by Toll-like receptors (TLRs), NOD-like receptors (NLRs), and other pattern-recognition receptors, thereby influencing intestinal immune homeostasis and tumor-associated immune states (38, 39). Under certain experimental conditions, sustained or relatively high-level exposure to LPS derived from Gram-negative bacteria can activate the TLR4/NF-κB signaling pathway in intestinal epithelial cells and myeloid immune cells and is associated with increased expression and release of inflammatory cytokines such as IL-1β, IL-6, and TNF-α (40, 41). It should be noted that the role of the NLRP3 inflammasome in CRC is not uniformly pro-inflammatory or tumor-promoting. Although NLRP3 activation can promote the maturation and release of IL-1β and IL-18 and participate in inflammatory responses, deletion of Nlrp3 or impairment of IL-18 signaling in AOM/DSS-induced models of colitis-associated cancer can instead exacerbate colitis and increase tumor formation, suggesting that the NLRP3–IL-18 axis may exert protective effects in specific contexts by maintaining epithelial barrier homeostasis and supporting tissue repair (42–44). Therefore, the biological effects of NLRP3 signaling are strongly dependent on cellular origin and disease context, and interpretation of its pro-inflammatory effects should also take into account the potentially distinct consequences of epithelial protective signaling and inflammasome activation within the tumor microenvironment.

In addition to LPS, microbial structural proteins and surface virulence factors may also participate in CRC-associated immune regulation. Certain bacterial flagellins can be recognized by TLR5 and activate downstream signaling, with experimental studies linking these effects to changes in intestinal epithelial cell proliferation and barrier function (45). In studies of Fusobacterium nucleatum, it is important to distinguish species-level associative observations from strain-, subspecies-, lineage-, or virulence factor-specific effects. Current mechanistic evidence suggests that some F. nucleatum-associated strains or subpopulations carrying specific virulence factors may influence CRC-related immune states through distinct pathways. FadA can bind to E-cadherin on the surface of intestinal epithelial cells and activate β-catenin-related signaling, whereas Fap2 can interact with the TIGIT receptor on NK cells and T cells and has been associated, under experimental conditions, with reduced cytotoxic immune function (46, 47).

Recent high-resolution genomic studies have further strengthened this lineage-specific perspective. A comparative genomic study published in 2024 showed that F. nucleatum subsp. animalis (Fna) can be further divided into two genetically distinct clades, Fna C1 and Fna C2. Among these, Fna C2, but not Fna C1, was significantly enriched in human CRC tumor tissues and exhibited stronger intestinal colonization and tumor-associated characteristics (48). This finding further indicates that conventional species-level classification of F. nucleatum may obscure subspecies- and lineage-level differences in ecological adaptation and pathogenic potential.

Accordingly, in this review, F. nucleatum is used primarily as an operational species-level designation that follows the taxonomic terminology adopted in the cited studies and does not presuppose specific reference to F. nucleatum sensu stricto, F. nucleatum sensu lato, the F. nucleatum species complex, or any particular subspecies. Only when the original study explicitly reports information on strains, subspecies, lineages, or virulence factors such as FadA or Fap2 do we use corresponding mechanism-specific descriptions. For studies with insufficient taxonomic or virulence factor resolution, findings are described uniformly as F. nucleatum-related observations or associations rather than being generalized as established strain-specific mechanisms. These findings should not be directly extrapolated to imply that all F. nucleatum strains exert uniform tumor-promoting or immune-evasive effects.

In addition, peptidoglycan and lipoteichoic acid derived from certain bacteria can influence macrophage and dendritic cell function through NOD1/NOD2- and TLR2-related signaling (30). Conversely, surface polysaccharides from some commensal bacteria may exert immunoregulatory effects. Polysaccharide A-related structural components have been shown in experimental studies to induce tolerance-associated signaling and modulate dendritic cell function, thereby limiting excessive inflammatory responses (49). In CRC-associated states, the immune effects generated by these structural components and surface virulence factors may be influenced by multiple factors, including strain characteristics, host barrier status, the local inflammatory environment, and the tumor microenvironment. Therefore, taxonomic enrichment of a microbial group should not be directly equated with a uniform functional effect or a definitive pathogenic mechanism.

#### 3.1.2 Microbial-related and host–microbiota co-metabolic signals

Unlike microbial structural components, which primarily engage pattern-recognition receptor-related signaling, microbiota-associated and host–microbiota co-metabolic signals may participate in CRC-related immune processes by modulating immune cell function, epithelial barrier status, tumor cell metabolism, and associated signaling pathways. Importantly, these signals are not necessarily produced by the gut microbiota alone. Rather, they may originate from microbial metabolism, host metabolism, tumor cell metabolism, immune cell metabolism, or the combined activities of multiple compartments, and their biological effects exhibit substantial source heterogeneity and context dependence. The major microbiota-associated and host–microbiota co-metabolic signals, their potential target cells, related pathways, evidence types, and possible roles in CRC immune regulation are summarized in **Table 1** (50–76).

**Table 1:** Microbiota-associated and host–microbiota co-metabolic signals involved in CRC-related immune regulation.

Metabolic signal	Biological source	Target cells	Major pathways/receptors	CRC-related immune effects	Evidence level	References
Acetate	Predominantly microbial fermentation product; also involved in host acetate metabolism	Intestinal epithelial cells, macrophages	FFAR2/GPR43, chemotaxis-related signaling	Maintains epithelial barrier integrity and modulates immune-cell recruitment	Human microbiome study; indirect mechanistic support	(50)
Propionate	Predominantly microbial-derived	Dendritic cells, Treg cells	FFAR2/3, HDAC inhibition, Foxp3	Promotes immune tolerance and myeloid-cell remodeling	Animal mechanistic study	(51)
Butyrate	Predominantly microbial-derived	CRC cells, CD8^+^ T cells	HDAC inhibition, FFAR2/3, Wnt/β-catenin	Context-dependent effects: anti-tumor immune regulation under physiological conditions; may differ under altered tumor metabolism (“butyrate paradox”)	Animal and mechanistic in vitro studies	(52, 53)
Primary bile acids	Host-derived hepatic bile acids; subsequently modified by gut microbiota	Intestinal epithelial cells, gut–liver axis	FXR signaling	Regulate bile acid pool, epithelial homeostasis, and microbial ecology	Human observational and animal studies	(54, 55)
Deoxycholic acid (DCA)	Host–microbial co-metabolite	CRC cells, macrophages	FXR/TGR5, ROS, DNA damage pathways	Associated with tumor-associated inflammation, oxidative stress, and metabolic adaptation	Animal and mechanistic in vitro studies	(56, 57)
Lithocholic acid (LCA)	Host–microbial co-metabolite	T cells, dendritic cells	VDR, TGR5	Regulates immune differentiation (including Th17/Treg balance) in a context-dependent manner	Indirect immunometabolic evidence; mainly non-CRC mechanistic studies	(58)
Ursodeoxycholic acid (UDCA)	Host bile acid pool; microbial conversion; exogenous supplementation depending on context	Intestinal epithelial cells	Oxidative stress-related pathways	May maintain epithelial homeostasis in specific experimental settings	Preclinical experimental evidence	(59)
Indole derivatives	Mainly microbial tryptophan metabolism with host–microbial interaction	Epithelial cells, DCs	AhR–IL-22 axis	Maintain barrier integrity and immune surveillance	Mechanistic animal and in vitro studies	(60)
Indole-3-propionic acid	Microbial-derived	Epithelial cells	AhR, PXR	Enhances epithelial barrier protection	Mechanistic evidence from experimental models; limited CRC-specific evidence	(61, 62)
Indole-3-acetic acid	Microbial-derived	Myeloid cells	AhR signaling	Context-dependent immune regulation	Experimental mechanistic evidence	(63)
Kynurenine	Mainly host-, tumor-, and immune-cell-derived tryptophan catabolism	T cells, NK cells, myeloid cells	IDO1/TDO–AhR pathway	Promotes immunosuppressive tumor microenvironment and immune escape	Human and animal tumor studies	(64)
Putrescine	Host and microbial metabolism	CRC cells	Cell-cycle regulation	Promotes epithelial proliferation and tumor growth in specific contexts	Animal and in vitro studies	(65)
Spermidine	Host and microbial metabolism	T cells, CRC cells	Autophagy, mitochondrial function	Regulates immune–metabolic homeostasis	Preclinical mechanistic evidence	(66, 67)
Spermine	Host and microbial metabolism	Myeloid cells	Oxidative-stress-related pathways	May promote immunosuppressive tumor microenvironment in experimental settings	Preclinical evidence	(68, 69)
Hydrogen sulfide (H_2_S)	Microbial and host metabolism	Epithelial cells	Mitochondrial metabolism, oxidative stress	Dual effects including epithelial injury or metabolic adaptation depending on concentration and context	Animal and in vitro studies	(70, 71)
Lactate	Tumor-cell-, stromal-cell-, immune-cell-, and microbiota-associated sources depending on context	CRC cells, NK cells, macrophages	HIF-1α, GPR81 signaling	Contributes to immunosuppressive tumor microenvironment and immune-metabolic reprogramming	Human and animal tumor studies	(72)
Succinate	Host and microbial metabolism; tissue- and disease-context dependent	Macrophages, epithelial cells	HIF-1α signaling, inflammatory metabolic reprogramming	Associated with myeloid activation and metabolic inflammation	Animal and mechanistic in vitro studies	(73)
TMAO	Host–microbial co-metabolite	Macrophages	Oxidative stress-related pathways	Associated with metabolic inflammation and immune modulation	Human observational evidence	(74)
Extracellular ATP	Released mainly from stressed, damaged, immune, stromal, and tumor cells	T cells, NK cells, macrophages	P2X7/P2Y receptors, NLRP3-related signaling	Functions as danger-associated signal and regulates inflammatory immune activation	Human and animal studies	(75)
Adenosine	Generated extracellularly from ATP through CD39/CD73 expressed by tumor, stromal, and immune cells	T cells, NK cells, myeloid cells	CD39–CD73–A2A/A2B receptor axis	Promotes immunosuppression, T-cell dysfunction, NK-cell inhibition, and immune escape	Human and animal tumor studies	(76)
Other metabolic and purinergic signals						
Lactate	Tumor-cell-, stromal-cell-, immune-cell-, and microbiota-associated sources depending on context	CRC cells, NK cells, macrophages	HIF-1α, GPR81 signaling	Contributes to immunosuppressive tumor microenvironment and immune-metabolic reprogramming	Human / animal	(72)
Succinate	Host + microbial metabolism; source depends on tissue and disease context	Macrophages, epithelial cells	HIF-1α signaling, inflammatory metabolic reprogramming	Associated with myeloid activation and metabolic inflammation	Animal / in vitro	(73)
TMAO	Host–microbial co-metabolite	Macrophages	Oxidative-stress-related pathways	Associated with metabolic inflammation and immune modulation	Human observational	(74)
Extracellular ATP	Mainly released by host, immune, stromal, and tumor cells under stress or cell damage	T cells, NK cells, macrophages	P2X7, P2Y receptors, NLRP3-related signaling	Acts as a danger-associated signal and may promote immune activation or inflammatory responses	Human / animal	(75)
Adenosine	Generated extracellularly from ATP by CD39/CD73 expressed on tumor, stromal, and immune cells	T cells, NK cells, myeloid cells	CD39–CD73–A2A/A2B receptor axis	Promotes immunosuppression, T-cell dysfunction, NK-cell inhibition, and immune escape	Human / animal	(76)

Short-chain fatty acids (SCFAs) are among the most extensively studied microbial metabolic signals in CRC. They are primarily generated through microbial fermentation of dietary fiber, although their effects within the local intestinal environment are also influenced by host absorption and utilization as well as the metabolic state of the tumor. Acetate, propionate, butyrate, and valerate can affect the functions of intestinal epithelial cells, dendritic cells, Treg cells, macrophages, and CD8^+^ T cells through mechanisms involving FFAR2/3, GPR43, and HDAC inhibition. Butyrate is particularly representative, and its biological effects are strongly dependent on cellular metabolic state and genetic background. Normal colonic epithelial cells can oxidize butyrate as an important energy substrate, whereas in CRC cells exhibiting Warburg-type metabolism, reduced butyrate oxidation leads to intracellular accumulation, allowing butyrate to act predominantly as an HDAC inhibitor and exert epigenetic effects associated with suppression of tumor cell proliferation (77). In contrast, Belcheva et al. showed in a compound-mutant mouse model that microbiota-derived butyrate could promote proliferation of abnormal colonic epithelial cells and enhance β-catenin-related activity, suggesting that butyrate may also contribute to tumorigenesis in specific Apc/Msh2 genetic contexts (78). Thus, the so-called butyrate paradox is unlikely to represent a simple dichotomy between protective and tumor-promoting effects; rather, it more likely reflects differences in the metabolic fate and biological actions of butyrate across distinct cellular metabolic states, host genetic backgrounds, and disease stages.

Bile acid-related signaling represents an important link among dietary patterns, host hepatobiliary metabolism, microbial transformation, and the CRC microenvironment. Primary bile acids are synthesized predominantly by the host liver and can influence the composition of the bile acid pool and the metabolic ecology of the gut microbiota. In contrast, secondary bile acids such as deoxycholic acid and lithocholic acid are largely generated through further microbial transformation of host-derived bile acids in the intestine and can act on intestinal epithelial cells, CRC cells, and immune cells through pathways involving FXR, TGR5, VDR, and ROS-related signaling. Deoxycholic acid, in particular, may be associated with DNA damage, tumor-associated immune remodeling, and metabolic adaptation of tumor cells. By comparison, certain bile acid-related signals may, under specific conditions, contribute to maintenance of epithelial homeostasis and restriction of tumor-promoting inflammatory responses. Thus, the effects of bile acids in CRC are strongly dependent on concentration, source, and microenvironmental context.

Tryptophan-related metabolic signals participate in CRC immune regulation mainly through pathways involving AhR, PXR, and IDO1/TDO–AhR signaling. Indole and its derivatives, indole-3-propionic acid, and indole-3-acetic acid are largely associated with microbiota-related tryptophan metabolism and have been linked in some experimental studies to barrier homeostasis, epithelial protection, and regulation of the local immune state. Kynurenine, however, is not a typical microbiota-derived metabolite and is produced predominantly through tryptophan catabolism in host cells, tumor cells, and immune cells. Enhanced kynurenine pathway activity is commonly associated with IDO1/TDO activation, suppression of T-cell function, reduced NK-cell activity, and increased immune checkpoint-related signaling and may contribute to the establishment of an immunosuppressive tumor microenvironment.

Polyamine-related metabolic signals may likewise participate in CRC-associated immunometabolic regulation. Putrescine, spermidine, and spermine can arise from combined host and microbial metabolism and can influence cell proliferation, DNA stability, autophagy, mitochondrial function, and immunometabolism. Moderate polyamine metabolism may support epithelial renewal and tissue repair, whereas abnormal accumulation may be associated with CRC cell proliferation, tumor metabolic adaptation, and maintenance of an immunosuppressive state. Sulfur-containing metabolic signals, such as hydrogen sulfide, also exhibit marked context dependence. They can influence mitochondrial metabolism, ROS levels, and DNA damage-related processes and may exert distinct effects, including barrier injury or metabolic adaptation, depending on their concentration and the surrounding microenvironment.

Beyond the signals described above, lactate, succinate, trimethylamine N-oxide (TMAO), and purinergic signaling also represent important components of metabolic–immune interactions in CRC. Lactate can be produced by tumor cells, stromal cells, immune cells, and certain microorganisms under different biological contexts and may contribute to the formation of an immunosuppressive tumor microenvironment through GPR81, HIF-1α, and immunometabolic reprogramming. Succinate likewise has both host and microbial sources, and its accumulation may contribute to shifts in myeloid cell function and inflammatory metabolic reprogramming. TMAO, by contrast, is a host–microbiota co-metabolite and may be associated with host metabolic abnormalities, oxidative stress, and tumor-associated immune remodeling.

Although ATP and adenosine belong to the same purinergic metabolic axis, they differ substantially in their sources, receptors, and immune effects. Extracellular ATP is primarily released by stressed, injured, or dying host cells, as well as by immune cells, stromal cells, and tumor cells, and can participate in danger signaling, inflammasome activation, and immune cell activation through receptors such as P2X7. In contrast, adenosine is generally generated through the stepwise hydrolysis of extracellular ATP by CD39 and CD73 and can suppress the antitumor functions of T cells, NK cells, and myeloid cells through A2A and A2B receptors, thereby contributing to immune evasion. Therefore, ATP and adenosine should not be simply grouped together as molecules with equivalent immune effects but should instead be discussed separately as purinergic signals with distinct, and in some contexts opposing, immunological directions.

These microbiota-associated and host–microbiota co-metabolic signals may also indirectly participate in tumor immune regulation by influencing adaptive immune cells, although such changes are generally built upon prior remodeling of the epithelial barrier, innate immunity, and myeloid cell function. Overall, CRC-associated metabolic–immune interactions are not driven solely by microbiota-derived metabolites but instead arise from the combined contributions of microbial metabolism, host metabolism, tumor metabolism, stromal cell metabolism, and immune cell metabolism. Their effects are highly dynamic and exhibit substantial heterogeneity in origin and context dependence. Further elucidation of the relationships between these metabolic signals and the CRC immune microenvironment may help identify precision intervention strategies and immunotherapy-sensitizing targets worthy of further validation.

#### 3.1.3 Immune effects of microbial secreted factors and nucleic acid-related molecules

In addition to microbial structural components and microbiota-associated metabolic signals, the gut microbiota can participate in CRC-related immune regulation through the secretion of various functional molecules. These include outer membrane vesicles (OMVs), secreted proteins and toxins, extracellular DNA/RNA, and quorum-sensing signals. OMVs are nanoscale vesicular structures actively released by Gram-negative bacteria and can carry biologically active components such as lipopolysaccharide, proteins, toxins, and nucleic acids (79). Some studies suggest that OMVs can cross the intestinal barrier and enter local tissues or even the circulation, thereby potentially influencing local or distant microenvironments (80). In addition, experimental studies have associated certain OMVs with reduced intestinal epithelial barrier stability, altered tumor cell proliferative states, and immune remodeling, although their direct mechanistic roles in human CRC remain to be further validated (81).

Microbial secreted proteins and toxins likewise represent important sources of signaling in CRC-associated microbiota–immune interactions. Bacteroides fragilis toxin (BFT), secreted by enterotoxigenic Bacteroides fragilis (ETBF), can cleave E-cadherin and activate β-catenin-, NF-κB-, and STAT3-related signaling (82). In Min mice, ETBF colonization induces STAT3 activation in colonic epithelial cells and subsets of infiltrating immune cells and elicits a pronounced Th17-biased inflammatory response; blockade of IL-17A or IL-23R markedly suppresses ETBF-induced colitis, epithelial hyperplasia, and tumor formation (83). This mechanism suggests that microbial virulence signals can remodel the epithelial and local innate immune environment and subsequently drive IL-23/Th17-related adaptive immune amplification, thereby linking microbial signals to tumor-promoting inflammatory responses.

This connection between innate and adaptive immunity is not limited to ETBF. Grivennikov et al. further showed in CRC models that adenoma formation is accompanied by loss of epithelial barrier integrity, allowing microbial-associated molecules to more readily enter tumor tissues and stimulate local myeloid cells to produce IL-23; IL-23 subsequently promotes IL-17-related immune responses that support tumor growth (84). Thus, the barrier disruption–microbial signal exposure–myeloid IL-23–IL-17/Th17 axis can be regarded as a representative mechanism through which microbiota-associated innate immune activation is amplified through adaptive immunity, consistent with the analytical framework of this review in which adaptive immune responses are positioned primarily downstream of innate immune and myeloid-cell remodeling.

Compared with the immune-regulatory mechanisms described above, colibactin produced by Escherichia coli carrying the pks pathogenicity island is supported by more direct evidence from human tumor genomes. Long-term exposure to pks^+^ E. coli can induce characteristic single-base substitution and insertion/deletion mutational patterns in human intestinal organoids, and corresponding colibactin-associated mutational features have subsequently been detected in human CRC genomes and classified as SBS88- and ID18-related mutational signatures (85–87). Colibactin is therefore not merely associated with DNA damage in experimental models but represents one of the few microbiota–CRC mechanisms in which exposure to a specific bacterial genotoxin, experimentally induced DNA damage patterns, and tumor-specific mutational signatures in humans can be directly linked. More recent large-scale whole-genome analyses of CRC have further shown that SBS88 and ID18 are more frequent in early-onset CRC and predominantly represent mutational events acquired early in tumor evolution (88). These findings provide relatively strong etiological and mechanistic evidence supporting a role for pks^+^ bacteria/colibactin in the development of a subset of CRCs, although they should not be interpreted to mean that all strains carrying the pks island inevitably cause CRC.

In addition, certain quorum-sensing molecules (QSMs) may regulate local inflammation, metabolism, and the tumor microenvironment through interactions with host receptors (89). Overall, the strength of evidence varies considerably among different microbial secreted factors: some mechanisms remain supported mainly by *in vitro* or animal studies, whereas colibactin-associated mutational signatures have received direct support at the level of human tumor genomics. Therefore, when evaluating the CRC-related effects of microbial secreted factors, it is important to distinguish experimental mechanistic evidence from molecular signature evidence that can be traced directly within human tumors.

#### 3.1.4 Fungal microbiome and innate immune interactions

The gut fungal microbiome may also participate in CRC-associated mucosal immune regulation. CRC-specific human studies have identified alterations in the composition and ecological networks of the gut mycobiome. Through multi-cohort mycobiome analyses, Coker et al. identified pronounced fungal dysbiosis and altered fungal ecological interactions in patients with CRC, suggesting that changes in the fungal microbiota may represent one component of CRC-associated microbial dysbiosis (90). However, these human studies currently provide mainly associative evidence and are insufficient to demonstrate that specific fungal alterations directly drive CRC-associated immune remodeling.

At the mechanistic level, fungal cell walls are rich in structural components such as α-glucans, β-glucans, and mannans (91). These molecules can be recognized by C-type lectin receptors (CLRs) expressed on innate immune cells, among which Dectin-1 and Mincle are important pattern-recognition receptors (92). Activation of CLR signaling can induce the expression of inflammation-related factors through the Syk–CARD9–NF-κB pathway and is associated with functional changes in macrophages and dendritic cells, thereby contributing to the regulation of mucosal immune responses (93). In the context of CRC, these mechanisms provide an experimental basis for understanding potential links between alterations in the fungal microbiome and local myeloid immune states, although their specific causal roles in human CRC remain to be further validated.

In addition, studies of Candida albicans suggest that its virulence factor candidalysin can damage epithelial cell membranes *in vitro* and in animal models and is associated with enhanced EGFR/MAPK signaling and NLRP3 inflammasome-related pathways (94–96). These changes may be accompanied by the release of inflammatory mediators such as IL-1β, epithelial barrier injury, and local inflammatory responses. However, this process is still supported primarily by evidence from experimental models, and its specific role in the initiation and progression of human CRC, as well as its strain specificity and context dependence, remains to be further clarified.

### 3.2 Regulation of gut microbial composition and spatial distribution by innate immunity

The innate immune system not only responds to signals derived from the gut microbiota but also participates in maintaining microbial composition, spatial distribution, and mucosal homeostasis. At the level of innate immunity, macrophages, neutrophils, and intestinal epithelial cells can restrict excessive bacterial growth and reduce microbial translocation through the release of reactive oxygen species (ROS), antimicrobial factors, and inflammatory mediators (97, 98). At the same time, sensing of microbial signals by pattern-recognition receptors may influence local oxygen tension, nutrient availability, and the metabolic environment and thereby be associated with changes in the ecological balance between obligate and facultative anaerobes (29).

The intestinal mucosal barrier is an important component through which innate immunity regulates the microbiota. The mucus layer secreted by intestinal epithelial cells not only forms a physical barrier but also restricts direct contact between the microbiota and epithelial tissues (99). When barrier integrity is preserved, most microorganisms are confined to the outer mucus layer. In some CRC tissues and relevant experimental models, however, abnormalities in mucus layer architecture and impaired spatial segregation have been observed, with some microbial populations detected within the inner mucus layer or even tumor tissues (100, 101). Such spatial remodeling may increase exposure to microbiota-associated signals and may be associated with alterations in the local immune state and tumor microenvironment.

Antimicrobial peptides and related antimicrobial effector molecules also play important roles in maintaining microbial homeostasis. Intestinal epithelial cells and Paneth cells can secrete antimicrobial molecules such as defensins, lysozyme, and Reg3 family proteins, although the antimicrobial spectra and ecological effects of these molecules are not identical. Reg3γ, in particular, is a secreted C-type lectin that recognizes bacterial peptidoglycan and preferentially restricts the proximity of Gram-positive bacteria to the intestinal epithelial surface, thereby maintaining spatial segregation between the microbiota and the host (102). Loss of Reg3γ allows Gram-positive commensal bacteria to localize closer to the small intestinal epithelium and is accompanied by enhanced mucosal immune responses, indicating that innate antimicrobial effectors can influence not only overall microbial abundance but also the spatial distribution of different microbial members through selective pressure (103). Accordingly, alterations in the expression patterns of antimicrobial molecules under CRC-associated conditions may exert asymmetric effects on different bacterial groups and thereby further contribute to remodeling of microbial composition and the spatial ecology of the mucosa (19, 104). In addition, although IgA coating represents an important component of mucosal adaptive immunity, it acts together with innate barrier mechanisms to restrict microbial adhesion and spatial invasion and may therefore participate in regulating microbial spatial distribution in CRC (105, 106).

Overall, the gut microbiota and the innate immune system may form complex bidirectional interactions through microbial structural components, metabolic signals, secreted factors, nucleic acid-related molecules, and host immune effector molecules. In CRC-related studies, microbiota-derived signals have been associated to varying degrees with pattern-recognition receptor activation, immunometabolic alterations, remodeling of myeloid cell function, and changes in NK-cell activity. Conversely, alterations in the mucosal barrier and innate immune state may also influence microbial colonization, spatial distribution, and metabolic function. Current evidence suggests that these interconnected changes may participate in remodeling of the tumor immune microenvironment, immune evasion, and other CRC-associated pathological processes. However, substantial differences remain in the types of evidence and the level of causal certainty across these mechanisms, and their precise causal direction and temporal sequence require further validation through longitudinal human studies and mechanistic investigations.

## 4 Dynamic evolution of gut microbiota–innate immune crosstalk during CRC initiation and progression

The initiation, local progression, and systemic dissemination of CRC involve complex interactions among tumor cell genetic alterations, mucosal barrier status, local immune responses, stromal remodeling, and vascular microenvironment changes. Current evidence suggests that gut microbial dysbiosis and innate immune abnormalities may participate in several of these processes; however, the sources of evidence and levels of causal certainty vary substantially among individual mechanisms. Some conclusions are primarily derived from animal models, *in vitro* experiments, or organoid-based studies, whereas others are supported by human observational investigations. Therefore, these findings should not be interpreted as fully established linear causal processes.

**Figure 1:**
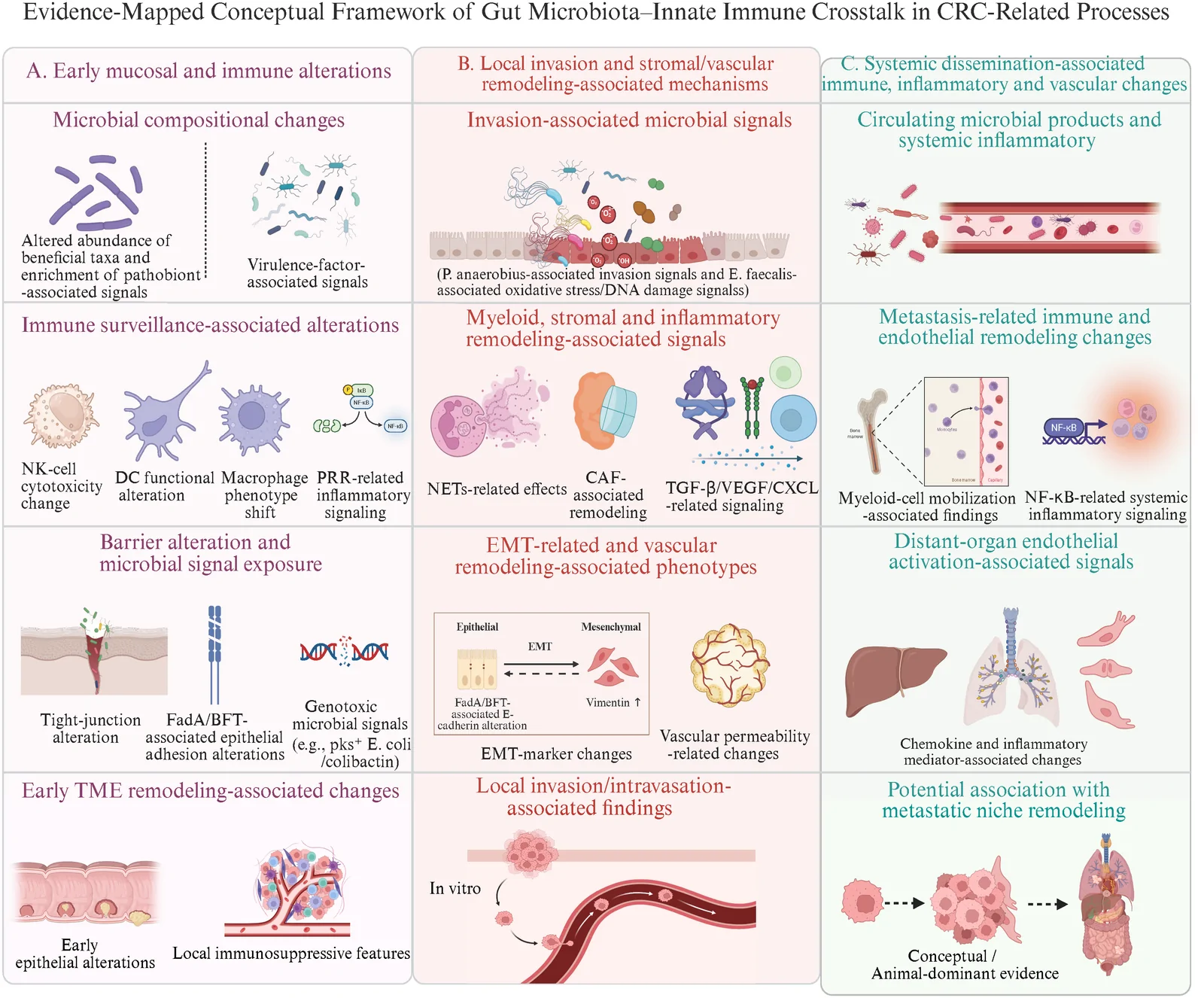
Evidence-mapped conceptual framework of gut microbiota–innate immune crosstalk in CRC-related processes. This image summarizes representative microbiota-associated signals and innate immune, stromal, and tumor microenvironment-related changes implicated in CRC-associated pathological processes. The three modules constitute an author-defined conceptual framework encompassing early mucosal and immune alterations, mechanisms associated with local invasion and stromal/vascular remodeling, and immune, inflammatory, and vascular changes related to systemic dissemination. This framework is not intended to represent a validated clinical or pathological staging system, nor does it imply a fixed temporal sequence or linear causal model of CRC progression. The illustrated relationships are derived from heterogeneous evidence sources, including human observational studies, human tissue analyses, animal models, in vitro experiments, and integrative mechanistic interpretation. Solid arrows indicate relationships supported by reported mechanistic evidence, whereas dashed arrows indicate indirect, context-dependent, or hypothesis-generating associations that require further validation. The evidence type, strength, and major limitations of the principal mechanisms illustrated here are further summarized in **Table 2**. CRC, colorectal cancer; DCs, dendritic cells; NK cells, natural killer cells; PRR, pattern recognition receptor; NF-κB, nuclear factor kappa B; TME, tumor microenvironment; pks^+^, polyketide synthase-positive; FadA, Fusobacterium adhesin A; BFT, Bacteroides fragilis toxin; NETs, neutrophil extracellular traps; CAF, cancer-associated fibroblast; TGF-β, transforming growth factor-β; VEGF, vascular endothelial growth factor; CXCLs, C-X-C motif chemokines; EMT, epithelial–mesenchymal transition.

Accordingly, this section integrates microbiota–innate immune interactions according to major CRC-associated pathological processes. **Figure 1** is presented as a conceptual evidence framework rather than a validated clinical or pathological staging system, and it does not represent a confirmed temporal sequence or linear causal model of CRC development. To improve the traceability between **Figure 1** and the mechanistic discussions, major microbiota–innate immune interactions illustrated in the figure are further mapped in **Table 2**, including their primary evidence sources, evidence strength, and major limitations.

**Table 2:** Evidence mapping of microbiota–innate immune crosstalk and related downstream CRC tumor microenvironment processes.

Module	Representative microbiota/signals	Host functional targets	Evidence type	Evidence strength & limitations	References
Altered immune surveillance–related changes	*Fusobacterium nucleatum* Fap2-positive subpopulations	NK cell TIGIT axis; cytotoxic immune function	In vitro; structural/mechanistic studies; limited human evidence	Moderate. The mechanism is virulence factor–specific and cannot be generalized to all *F. nucleatum* strains; direct causal evidence in human CRC remains limited	(107, 108)
Epithelial signaling alteration–related mechanisms	*F. nucleatum* FadA-associated signaling	E-cadherin; β-catenin pathway; intestinal epithelial cells	In vitro; animal models; partial human tissue correlation	Moderate. Evidence supports association with epithelial signaling dysregulation, but its independent driving role in early human CRC remains unverified	(109)
DNA damage–associated alterations	pks^+^ *Escherichia coli*/colibactin	Epithelial cells; DNA damage response; genomic stability	In vitro; organoid/ex vivo models; animal models; partial human genomic evidence	Moderate to strong. Experimental evidence is robust, but complete causal chains in human CRC remain to be confirmed longitudinally	(85, 86)
Barrier dysfunction & microbial signal exposure	LPS, flagellin, bacterial DNA and other MAMPs	Mucus layer; tight junctions; TLRs/NLRs/cGAS–STING pathways	Human observational; animal models; in vitro	Moderate. Supports associations between dysbiosis, barrier disruption, and immune activation, but temporal and causal directions remain unclear	(110, 111)
Epithelial barrier & immune modulation–related alterations	Enterotoxigenic *Bacteroides fragilis*(ETBF)/BFT toxin	E-cadherin; β-catenin; STAT3; local myeloid cells	In vitro; animal models; human association studies	Moderate. Mechanisms are relatively well supported experimentally, but human causal strength remains uncertain	(83, 112)
Myeloid inflammation–related alterations	Parasutterella excrementihominis-associated inflammatory signals and other microbiota-derived inflammatory stimuli	Macrophages; dendritic cells; TLR2/4–MyD88–NF-κB pathway	Animal models; in vitro; partial human evidence	Moderate. Primarily supports effects on myeloid activation and pro-inflammatory signaling; direct human mechanistic evidence remains limited	(112, 113)
Inflammation-induced DNA damage–related changes	Neutrophil-driven inflammation; indirect microbial inflammatory signals	Neutrophils; ROS/RNS; DNA damage response	Animal models; in vitro	Moderate to low. Not microbiota-specific; largely reflects inflammation–genomic instability coupling	(114, 115)
Immunometabolic alterations	Secondary bile acid-related host–microbiota co-metabolic signals (e.g., deoxycholic acid)	FXR/TGR5 signaling; ROS-related responses; epithelial and myeloid cells	Human observational; animal models; in vitro	Moderate. Effects are highly concentration- and context-dependent; cannot be simplified into purely pro- or anti-tumor roles	(116, 117)
EMT & local invasion–related alterations	*P. anaerobius*; pks^+^ *E. coli*/colibactin; virulence/metabolic signals	CRC cells; EMT markers; MMP-2/MMP-9; NF-κB/PI3K–Akt pathways	In vitro; animal models	Moderate to low. Evidence supports association with invasive phenotypes, but not independent EMT drivers in human CRC	(12, 116)
Stromal remodeling & vascular microenvironment changes	Microbial inflammatory signals; LPS; flagellin	TAMs; neutrophils; CAFs; VEGF; CXCLs; Ang2; MMPs	Animal models; in vitro; limited human observational evidence	Moderate to low. Mostly indirect or preclinical evidence; cannot directly infer promotion of intravasation	(118, 119)
NETs & circulating survival–related changes	Inflammation-associated microbial signals (indirect)	Neutrophils; NETs; circulating tumor cell adhesion/survival	Animal models; in vitro	Low to moderate. NET–tumor interaction supported experimentally, but microbiota specificity and human causality remain insufficient	(120, 121)
Putative metastatic niche–related alterations	Circulating microbial metabolites; systemic inflammatory signals; co-metabolic factors	Endothelial cells; myeloid cells; chemokine networks; liver/lung microenvironment	Animal models; human observational; conceptual integration	Low. Primarily hypothesis-driven and indirect evidence; should not be interpreted as proven metastatic colonization mechanism	(122, 123)

### 4.1 CRC initiation stage

#### 4.1.1 Local immune surveillance and shaping of the myeloid microenvironment

In the normal intestinal mucosa, the innate immune system contributes to the maintenance of local immune homeostasis and the restriction of abnormal cell expansion, although the roles of different cell populations are not identical. NK cells can participate in immune surveillance of abnormal epithelial cells through direct cytotoxic activity, whereas macrophages and dendritic cells more commonly influence the local immune environment through phagocytic clearance, pattern recognition, antigen presentation, and regulation of inflammatory mediators (124, 125). Thus, the role of innate immunity in CRC-associated states encompasses both direct surveillance of abnormal cells and the shaping of pro-inflammatory, immunosuppressive, or antitumor microenvironments.

These effects are also influenced by the molecular and immune context of CRC. As discussed above, CMS1/MSI-H tumors generally exhibit more prominent immune activation and immune cell infiltration, whereas MSS/pMMR tumors, which account for the majority of CRC cases, overall display weaker T-cell-inflamed immune features (9). Therefore, tumor immune surveillance in its classical sense cannot be assumed to operate uniformly across all CRC subtypes. In relatively less immunoreactive contexts such as MSS/pMMR CRC, microbiota-associated signals may be more likely to alter the myeloid immune environment by modulating macrophage functional states, dendritic cell activation, and the accumulation of myeloid-derived suppressor cells, thereby generating local niches that may either favor or constrain tumor development (126). At present, sufficient longitudinal human evidence defining the temporal sequence among microbial alterations, myeloid remodeling, and tumor development remains lacking, and causal relationships are inferred mainly from animal models, *in vitro* experiments, and associative studies of human tissues.

Studies of Fusobacterium nucleatum further illustrate the distinction between suppression of immune surveillance and shaping of the myeloid microenvironment. F. nucleatum subpopulations expressing Fap2 can interact with the TIGIT receptor on NK cells and T cells and suppress cytotoxic immune activity under experimental conditions, representing a relatively direct mechanism of impaired tumor immune surveillance (107). By contrast, other F. nucleatum-associated signals can engage TLR4-dependent inflammatory pathways. In CRC cells, F. nucleatum has been shown to activate TLR4/MYD88/NF-κB signaling, whereas in macrophages, F. nucleatum can promote M2-like polarization through TLR4-dependent pathways, including IL-6/STAT3/c-MYC and TLR4/NF-κB/S100A9 signaling (127, 128). These effects are therefore more appropriately interpreted as remodeling of the local myeloid and inflammatory microenvironment rather than as a direct impairment of tumor-cell clearance.

Similarly, enterotoxigenic Bacteroides fragilis, namely specific B. fragilis strains carrying BFT, can influence epithelial cells and the local immune environment through E-cadherin/β-catenin, STAT3, and related inflammatory signaling and can further be linked to IL-23/Th17-associated adaptive immune amplification (87, 129). Accordingly, microbiota–immune interactions in CRC should not be uniformly summarized as enhancement or suppression of immune surveillance. Instead, a distinction should be made between two different but interconnected mechanistic levels: direct cytotoxic immune surveillance and shaping of the myeloid immune microenvironment. Differences in CRC molecular subtype, microbial strain characteristics, and host immune background may further determine the relative importance of these two processes.

#### 4.1.2 Barrier dysfunction and microbial signal exposure

The intestinal mucosal barrier is composed of the mucus layer, epithelial cells, tight-junction structures, and the local immune system and plays a critical role in maintaining spatial segregation of the microbiota and limiting the exposure of host tissues to microbiota-associated molecules. In CRC-related studies, alterations in microbial composition have been associated to some extent with abnormalities in barrier structure and may be accompanied by increased intestinal permeability, although findings and causal directions are not entirely consistent across studies (130).

Unlike changes involving immune cell function alone, barrier dysfunction is primarily characterized by impaired epithelial structural integrity, altered stability of the mucus layer, and weakened mucosal defense capacity. Some experimental studies suggest that microbial alterations may affect goblet cell function and mucus secretion and are associated with reduced MUC2 expression and thinning of the mucus layer (131, 132). In addition, certain pro-inflammatory microbiota-associated signals and microbial metabolic signals can affect the expression of tight-junction proteins such as ZO-1, Occludin, and Claudins in experimental models and are thereby associated with impaired epithelial barrier function (133).

As barrier integrity declines, microbiota-associated molecules such as LPS, flagellin, and bacterial DNA may more readily enter the lamina propria and become associated with activation of pathways involving TLRs, NLRs, and cGAS–STING (134). However, direct evidence demonstrating that this process constitutes a defined initiating event in human CRC remains limited. Current evidence is therefore more appropriately interpreted as supporting interrelated changes among microbial dysbiosis, barrier disruption, and local immune activation rather than a unidirectional linear causal chain.

#### 4.1.3 Association of low-grade inflammation with the formation of the tumor-associated immune microenvironment

Persistent low-grade inflammation, alterations in microbial ecology, and dysregulated local immune responses are considered to be associated with the formation of the tumor-associated immune microenvironment in CRC. However, current understanding of the temporal sequence and causal direction of these changes during early CRC development is still derived mainly from animal models, *in vitro* studies, and cross-sectional human investigations, and consistent longitudinal mechanistic evidence in humans remains lacking.

In experimental models, Peptostreptococcus anaerobius has been reported to influence myeloid cell functional states through TLR2/4-, MyD88-, and NF-κB-related signaling and has been associated with increased expression of pro-inflammatory and tumor-associated molecules (135, 136). In addition, neutrophils can release ROS and reactive nitrogen species in some experimental settings. These effects are associated with DNA damage and genomic instability and may contribute to adenoma formation or early tumor-associated alterations, although the relevant evidence is derived primarily from animal models (137).

The IL-23/IL-17 axis driven by microbial signal exposure following barrier disruption represents a characteristic mechanism linking microbiota exposure, myeloid inflammation, and tumor growth. Grivennikov et al. showed that colorectal adenoma formation can be accompanied by impaired epithelial barrier integrity, allowing microbiota-associated products to enter tumor regions and stimulate tumor-associated myeloid cells to produce IL-23. IL-23 subsequently promotes local IL-17-related immune responses and supports tumor growth and progression (84). This process indicates that microbiota-associated low-grade inflammation is not simply reflected by increased levels of inflammatory mediators but may form a sustained tumor-promoting inflammatory circuit through barrier disruption, microbial signal exposure, myeloid IL-23 production, and downstream IL-17-related immune amplification.

In addition, diet-related factors, such as high-fat diet-induced alterations in the microbiota and bile acid metabolism, may influence the local tumor immune state and epithelial metabolic adaptation through FXR, TGR5, and oxidative stress-related pathways (57, 61). Some CRC mouse models have also shown that microbial dysbiosis can be accompanied by myeloid cell accumulation, STAT3 activation, and tumor formation, whereas restricting the expansion of specific Enterobacteriaceae can reduce tumor burden and improve the local immune state under experimental conditions (130, 138). Collectively, these findings suggest that microbiota-associated signals may contribute to the formation of a tumor-related inflammatory microenvironment through the combined effects of barrier disruption, remodeling of myeloid cell function, and downstream immune amplification. However, their temporal dynamics and causal strength in human CRC remain to be further established.

### 4.2 CRC progression stage

#### 4.2.1 Local invasion-related phenotypes and EMT-associated signaling

Local invasion in CRC involves complex interactions among tumor cells, stromal cells, immune cells, and the extracellular matrix. Epithelial–mesenchymal transition (EMT) is an important biological program associated with tumor cell migration and invasion and is characterized by reduced expression of epithelial markers such as E-cadherin, together with alterations in mesenchymal-associated molecules including vimentin, N-cadherin, Snail, Slug, and Twist (139–141).

Microbiota-associated signals may be linked to EMT-related phenotypes and local invasion, although the current evidence is derived mainly from *in vitro* experiments and animal models. In these studies, Peptostreptococcus anaerobius has been reported to influence CRC cell proliferation, migration, and invasion-related phenotypes through signaling pathways such as PI3K–Akt and NF-κB (142). Escherichia coli carrying the pks island can produce colibactin and induce DNA damage and genomic instability in experimental systems, thereby being associated with the accumulation of invasion-related phenotypes (143). In some experimental studies, Enterococcus faecalis has also been observed to induce ROS generation and cellular stress responses and may influence cell adhesion and local infiltration processes (144).

In addition, certain microbiota-associated metabolic signals and virulence factors have been associated in experimental models with increased expression of matrix metalloproteinases such as MMP-2 and MMP-9, potentially contributing to extracellular matrix degradation and alterations in tissue barriers (145). However, it should be emphasized that these findings primarily support experimental associations between microbiota-associated signals and invasive phenotypes and do not directly demonstrate that such signals independently drive EMT or local invasion in human CRC. Local invasion also involves tumor-associated macrophages, neutrophils, cancer-associated fibroblasts, and other stromal components, which release TGF-β, VEGF, MMPs, and multiple chemokines and collectively contribute to matrix remodeling and local infiltration (146).

#### 4.2.2 Intravasation, the vascular microenvironment, and changes associated with systemic dissemination

Intravasation refers to the entry of locally invasive tumor cells into blood vessels or lymphatic structures and represents an important step in systemic tumor dissemination (147). However, whether microbiota–innate immune interactions directly participate in intravasation and the formation of distant metastases in CRC remains insufficiently supported by human mechanistic evidence. Current mechanistic interpretations are derived mainly from animal models, *in vitro* experiments, and indirect inference from studies of the tumor microenvironment.

Available experimental evidence suggests that LPS, flagellin, and other microbiota-associated molecules can influence the functional states of macrophages, neutrophils, and dendritic cells and are associated with pro-angiogenic signaling, inflammatory chemotaxis, and alterations in the vascular microenvironment. These changes may be accompanied by increased expression of factors such as VEGF, CXCL1, CXCL8, and Ang2 and may occur together with angiogenesis and abnormal vascular architecture in model systems (148, 149). However, these findings should not be interpreted as evidence that microbial dysbiosis directly promotes the entry of CRC cells into the circulation.

Immune cells may also participate in remodeling of the perivascular microenvironment. In some studies, macrophages can influence basement membrane and endothelial structures through the secretion of MMPs, TGF-β, and VEGF (150), whereas neutrophil extracellular traps (NETs) have been associated in experimental models with enhanced tumor cell adhesion and survival in the circulation (151). Different microbial taxa or microbiota-associated signals may show associations in different directions during these processes. For example, some F. nucleatum-related observations and enterotoxigenic Bacteroides fragilis, namely specific B. fragilis strains carrying BFT, have been associated in certain experimental models with pro-angiogenic activity, inflammatory remodeling, and immunosuppressive states. By contrast, certain commensal bacteria, including Bifidobacterium and Lactobacillus, have been associated with maintenance of barrier homeostasis and regulation of inflammation in specific experimental contexts (152–154). Nevertheless, these findings remain derived predominantly from model studies or indirect evidence and should not be simply extrapolated to uniform metastasis-promoting or protective effects at the species level.

## 5 Advances in gut microbiota–based clinical interventions and immunomodulatory strategies

**Figure 2:**
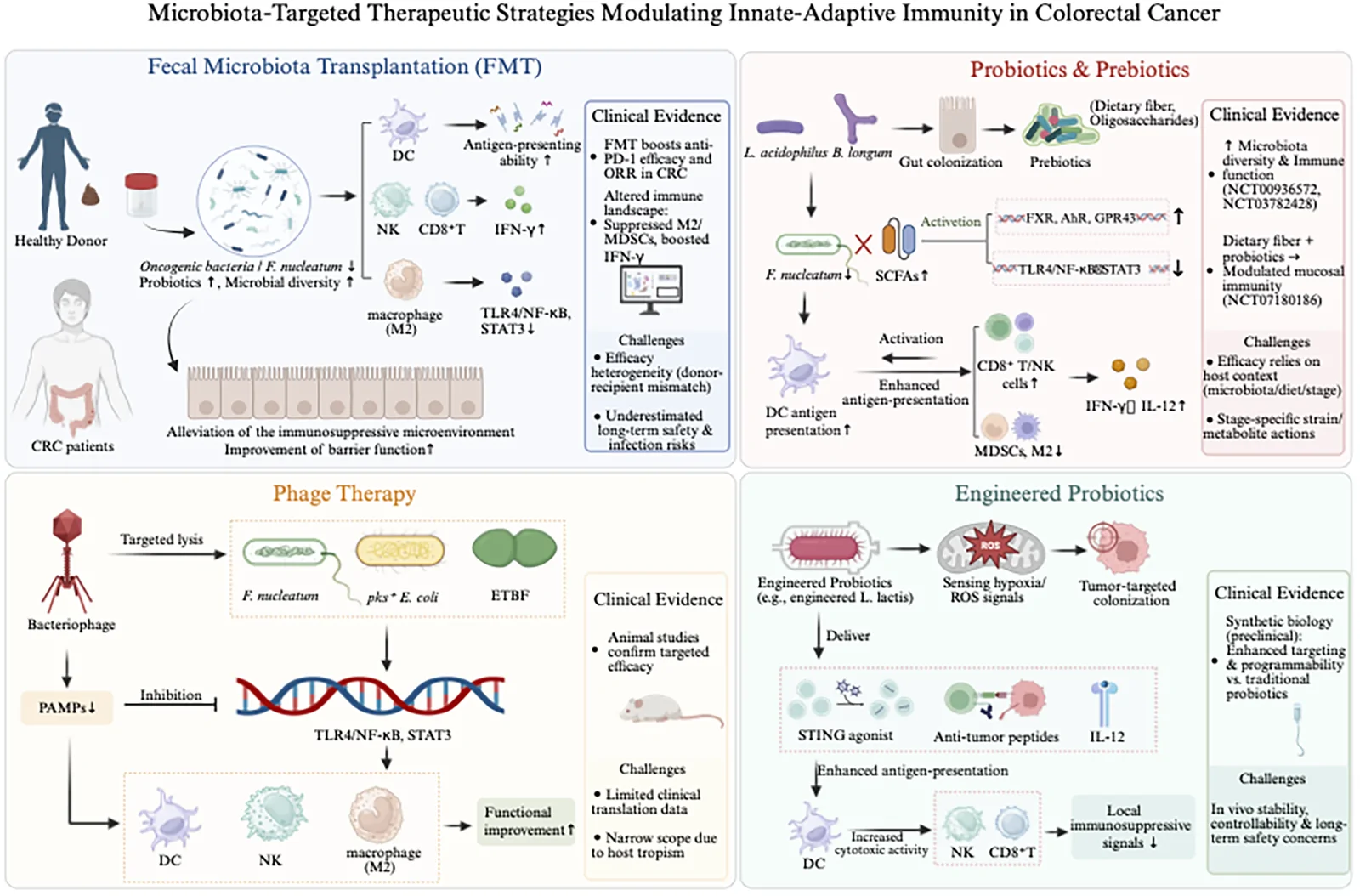
Potential microbiota-targeted therapeutic strategies and their proposed immunomodulatory effects in CRC. This image summarizes major microbiota-targeted strategies currently being investigated for their potential to modify CRC-associated microbial ecosystems, barrier function, and immune responses. Fecal microbiota transplantation (FMT) aims to restore or reshape microbial community structure and metabolic function and may influence dendritic-cell activity, NK-cell function, CD8^+^ T-cell responses, and myeloid-cell states. Cross-cancer clinical observations linking gut dysbiosis with immune checkpoint inhibitor response provide additional rationale for investigating FMT in combination with immunotherapy, although its efficacy in CRC remains under clinical evaluation. Probiotics and prebiotics may alter microbial composition and metabolite production, including short-chain fatty acids (SCFAs), with potential downstream effects on barrier integrity, antigen presentation, inflammatory signaling, and immune-cell function. Phage-based approaches seek to selectively target potentially tumor-associated bacterial populations, including Fusobacterium nucleatum-associated taxa, pks^+^ Escherichia coli, and enterotoxigenic Bacteroides fragilis, with potential downstream effects on bacteria-associated inflammatory and immune pathways. Engineered probiotics represent emerging programmable platforms for localized delivery of immunomodulatory molecules, including STING agonists, IL-12, and antitumor peptides, and may enhance antigen presentation and cytotoxic immune activity within the tumor microenvironment. However, most of these proposed immunomodulatory effects are currently supported predominantly by preclinical studies, mechanistic investigations, or early exploratory clinical studies. Their therapeutic efficacy, safety, reproducibility, long-term stability, and controllability in CRC require further prospective validation. CRC, colorectal cancer; FMT, fecal microbiota transplantation; DC, dendritic cell; NK, natural killer; SCFAs, short-chain fatty acids; MDSCs, myeloid-derived suppressor cells; TLR4, Toll-like receptor 4; NF-κB, nuclear factor kappa B; STAT3, signal transducer and activator of transcription 3; FXR, farnesoid X receptor; AhR, aryl hydrocarbon receptor; GPR43, G protein-coupled receptor 43; PAMPs, pathogen-associated molecular patterns; STING, stimulator of interferon genes; IL-12, interleukin-12; IFN-γ, interferon-γ; ROS, reactive oxygen species; ETBF, enterotoxigenic Bacteroides fragilis; pks^+^, polyketide synthase-positive.

The gut microbiota and the host innate immune system engage in complex and dynamic interactions, and disruption of this balance is considered to be closely associated with CRC development, immune evasion, and remodeling of the tumor microenvironment. Current microbiota-based intervention strategies mainly include fecal microbiota transplantation (FMT), probiotics, prebiotics, and emerging microbiota-modulating approaches (**Table 3**). It should be noted that most of the studies listed in **Table 3** are registered or ongoing clinical trials designed primarily to evaluate potential intervention effects, and sufficiently published results demonstrating definitive clinical benefit are currently lacking. Current microbiota-related intervention strategies and their potential immunomodulatory effects are illustrated in **Figure 2**.

**Table 3:** Gut microbiota-based clinical intervention studies in CRC.

Intervention type	Trial ID	Study population	Phase	Sample size	Recruitment status	Intervention	Comparator	Primary outcome	Results availability
FMT	NCT04729322	Anti-PD-1 non-responders with metastatic CRC or related dMMR/MSI-H solid tumors	Phase 2	15	Active, not recruiting	Antibiotic pretreatment followed by FMT capsule combined with anti-PD-1 re-introduction using nivolumab or pembrolizumab	No separate comparator/control group	Objective response rate	Exploratory small-sample study; preliminary results are available, but should not be interpreted as established clinical efficacy
FMT	NCT05279677	Patients with unresectable locally advanced or metastatic MSS/pMMR CRC who had failed standard treatment and had not previously received immune-checkpoint inhibitors or TKIs	Phase 2	30	Unknown status; last known status was recruiting, with the record last verified in March 2022	FMT capsules combined with sintilimab and fruquintinib as later-line treatment	No separate comparator/control group; single-arm, open-label study	Objective response rate, assessed for up to 2 years	No results posted; registered single-arm exploratory phase II study with an outdated recruitment record, not evidence of established clinical efficacy
FMT	NCT07509398	Patients with initially unresectable locally advanced or metastatic CRC who have not received first-line standard therapy	Phase 2/3	220	Not yet recruiting	FMT combined with standard first-line systemic therapy, including chemotherapy with or without targeted or anti-angiogenic agents	Standard first-line systemic therapy alone	Objective response rate	No results posted; registered ongoing/planned trial, not evidence of established clinical efficacy
FMT	NCT07486492	Patients with microsatellite stable metastatic CRC who progressed after first-line chemotherapy and targeted therapy	Early Phase 1	10	Not yet recruiting	Young-donor FMT combined with PD-1 inhibitor immunotherapy and FOLFIRI chemotherapy	No separate comparator/control group	Incidence of SAEs, TRAEs, and intervention adjustments due to adverse events	No results posted; exploratory safety-focused study, not evidence of established clinical efficacy
Probiotics	NCT00936572	Patients with CRC undergoing elective colorectal surgery	Phase 2	35	Completed	Perioperative oral probiotics containing La1 and BB536 at high or low dose	Placebo/maltodextrin	Morphological and microbiological evaluation of colonic microflora and gastrointestinal function	No results posted on ClinicalTrials.gov; completed perioperative microbiome/immune-modulation study, not evidence of direct antitumor efficacy
Probiotics	NCT03782428	Patients with CRC planned for colorectal resection	Not Applicable	52	Completed	Probiotic supplementation containing six Lactobacillus and Bifidobacterium strains twice daily for six months after surgery	Placebo	Changes in circulating inflammatory cytokines, including TNF-α, IFN-γ, IL-6, IL-10, IL-12, IL-17A, IL-17C, and IL-22	No results posted on ClinicalTrials.gov; completed supportive-care trial evaluating inflammatory cytokine changes, not evidence of direct antitumor efficacy
Dietary fiber + probiotics	NCT07194954	Malnourished patients with stage II–IV/advanced CRC after resection and receiving active adjuvant chemotherapy	Not Applicable	80	Completed	Standard nutritional support plus daily mixed dietary fiber and multi-strain probiotics for 12 weeks	Standard nutritional support alone	Changes in IgA, IgG, CD4+/CD8+ ratio, gut microbiota alpha diversity, and relative abundance of probiotic genera	No results posted on ClinicalTrials.gov; completed nutritional-support and microbiome/immune-modulation study, not evidence of direct antitumor efficacy
EPA-related microbiome study	NCT04682665	Patients with colorectal cancer liver metastases already enrolled in the EMT2 trial	Observational	81	Completed	Biospecimen collection from patients receiving icosapent ethyl/EPA-EE in the EMT2 trial	Placebo group in the parent EMT2 trial	Changes in gut microbiome taxa, microbial gene expression, lipid mediators, immune cell populations, immune checkpoint regulators, and their relationship with survival	No results posted on ClinicalTrials.gov; completed prospective biospecimen/mechanistic study, not direct evidence of prebiotic or antitumor efficacy

### 5.1 Fecal microbiota transplantation

FMT is currently one of the more intensively investigated translational strategies for microbiota modulation in CRC. Its central principle is to transplant the intestinal microbial community from rigorously screened donors in an attempt to reshape the recipient microbiota and its metabolic and immunoregulatory functions. Clinical exploration of FMT in CRC has mainly focused on combination with standard systemic therapy, immune checkpoint inhibitors (ICIs), and immunotherapy rechallenge. Ongoing registered studies are evaluating the safety and preliminary efficacy of FMT combined with standard first-line systemic therapy in initially unresectable locally advanced or metastatic CRC. In addition, a single-arm phase II study is investigating FMT combined with sintilimab and fruquintinib as later-line therapy for advanced CRC, while small-scale studies are assessing the feasibility of FMT combined with PD-1 inhibitor rechallenge or with a PD-1 inhibitor plus FOLFIRI chemotherapy (**Table 3**). However, most of these studies are not yet recruiting, ongoing, or still at an early exploratory stage, and some use single-arm, small-sample designs or primarily evaluate safety and feasibility. Therefore, current evidence remains insufficient to support a definitive conclusion that FMT provides clear antitumor clinical benefit in CRC.

Clinical exploration of FMT combined with ICIs has also been driven by studies of microbiota–immunotherapy interactions in other tumor types. Routy et al. found that, among patients with advanced cancers receiving PD-1/PD-L1 inhibitors, antibiotic exposure was associated with poorer immunotherapy outcomes, whereas the presence of specific microbial taxa, including Akkermansia muciniphila, was associated with better treatment responses (155). Gopalakrishnan et al. similarly observed marked differences in gut microbial composition and diversity between responders and nonresponders among patients with melanoma treated with anti-PD-1 therapy (156). Subsequent early clinical studies showed that FMT derived from immunotherapy responders, when administered to patients with melanoma who had previously developed resistance to anti-PD-1 therapy, could reshape the gut microbiota and tumor immune microenvironment in some patients and restore a degree of responsiveness to anti-PD-1 treatment (157, 158). Because these studies were conducted primarily in non-CRC malignancies, their clinical efficacy cannot be directly extrapolated to CRC. Nevertheless, together they support the concept that the gut microbiota may influence ICI responsiveness and provide an important cross-tumor rationale for clinical investigation of FMT combined with immunotherapy in CRC.

From a mechanistic perspective, the potential effects of FMT are unlikely to arise from a single microbial species or metabolite but may instead involve coordinated remodeling of microbial ecology, metabolic signaling, and host immune status. Animal and experimental studies suggest that FMT can alter short-chain fatty acids, bile acids, and other microbiota-associated metabolic signals and subsequently influence the functional states of CD8^+^ T cells, NK cells, dendritic cells, and myeloid cells (159). Some model studies have also associated FMT with reduced abundance of tumor-promoting microbial taxa, attenuation of inflammatory signaling pathways such as TLR4/NF-κB and STAT3, and alterations in the composition of tumor-associated macrophages and myeloid-derived suppressor cells (160–162). Therefore, the potential immunosensitizing effect of combining FMT with ICIs is more likely to involve multilayered interactions among restoration of microbial ecology, adjustment of the metabolic environment, remodeling of innate/myeloid immunity, and downstream antitumor immune responses, rather than simply reflecting an increase in a particular “beneficial” bacterium or metabolite.

Importantly, the clinical translation of FMT in CRC remains subject to substantial heterogeneity. Donor selection criteria, baseline recipient microbiota, prior exposure to antibiotics and anticancer therapies, tumor molecular subtype, disease stage, route and frequency of administration, and the choice of combination regimen may all influence treatment outcomes. In CRC in particular, MSI-H/dMMR and MSS/pMMR tumors have distinct immune backgrounds, and it remains unclear whether FMT can produce consistent immunomodulatory effects across different molecular subtypes. In addition, long-term safety, potential transmission of pathogens, transfer of antimicrobial resistance genes, abnormal immune activation, and issues related to standardized preparation and quality control require further evaluation in prospective clinical studies. Overall, FMT combined with immunotherapy has a clear biological rationale and supporting clinical evidence across tumor types, but the responsive patient populations, mechanisms of action, and boundaries of clinical benefit in CRC remain to be defined through prospectively designed studies incorporating appropriate patient stratification.

### 5.2 Probiotics, dietary fiber, and EPA-related microbiota modulation

Probiotic- and dietary fiber-related interventions are among the more commonly investigated supportive strategies for microbiota modulation in CRC. Current clinical studies have focused mainly on perioperative management, postoperative recovery, nutritional support, and changes in inflammatory or immune-related indicators rather than on direct antitumor treatment. For example, some completed studies have evaluated the effects of perioperative probiotics on colonic mucosal microbial colonization, gastrointestinal function, immune-inflammatory responses, and bacterial translocation. In addition, randomized, double-blind, placebo-controlled studies have examined changes in circulating inflammatory mediators following long-term supplementation with formulations containing Lactobacillus and Bifidobacterium in postoperative patients with CRC. More recent registered studies have also explored dietary fiber combined with multi-strain probiotics in malnourished patients with advanced CRC, focusing primarily on outcomes such as IgA, IgG, the CD4^+^/CD8^+^ ratio, microbial diversity, nutritional status, and quality of life (**Table 3**). Therefore, the current clinical evidence is more appropriately interpreted as supportive research on modulation of the microbiota and immune status rather than as direct evidence that probiotics or dietary fiber provide established antitumor efficacy.

Mechanistic studies suggest that commensal bacteria may be associated with regulation of CD8^+^ T-cell and NK-cell function and can influence the expression of immune-related factors such as IFN-γ and IL-12 in experimental models (163, 164). In addition, some probiotic interventions have been associated in animal models with alterations in the infiltration patterns of MDSCs, M2-like macrophages, and inflammatory cells within tumor tissues (165). Dietary fiber, in turn, can serve as a metabolic substrate for the gut microbiota and promote the production of metabolites such as short-chain fatty acids, thereby indirectly influencing epithelial barrier function, myeloid cell activity, and local inflammatory responses (165).

Beyond conventional probiotics and dietary fiber, studies of EPA-related microbiota mechanisms have provided an additional perspective on microbiota–immune regulation in CRC liver metastasis. Relevant prospective biospecimen studies are ongoing or have investigated gut microbial composition, microbial gene expression, lipid mediators, immune cell populations, and immune checkpoint molecules in patients with CRC liver metastases in the context of EPA exposure or treatment (**Table 3**). However, these studies are more appropriately considered mechanistic and biomarker-oriented investigations and should not be simply classified as clinical efficacy evidence for conventional prebiotics. Overall, probiotics, dietary fiber, and metabolism-related microbiota-modulating strategies have translational potential, but their effects are influenced by multiple factors, including baseline host microbiota, nutritional status, disease stage, treatment context, and the immune microenvironment. Larger prospective studies incorporating patient stratification and clinically relevant outcomes are therefore still required.

### 5.3 Emerging microbiota-targeted intervention strategies

With advances in microbiome medicine and synthetic biology, microbiota-targeted strategies for CRC are gradually shifting from broad ecological modulation toward more precise and targeted interventions. Phage therapy represents a highly specific microbial intervention capable of selectively lysing defined bacterial targets and may therefore provide a potential strategy for targeting CRC-associated tumor-promoting bacteria (166). However, most relevant studies remain at the preclinical or early exploratory stage, and clinical evidence is still limited.

Engineered probiotics represent a further transition from ecological modulation toward functional delivery. Through genetic engineering, these strategies can be designed to express therapeutic molecules in a targeted manner, for example by releasing immunomodulatory factors in response to stimuli within the tumor microenvironment. In model studies, such approaches have shown potential effects on the functions of CD8^+^ T cells, NK cells, and dendritic cells (167–169).

In addition, CRISPR-based microbiota editing and personalized microbiome modulation are increasingly emerging as areas of research interest. Integration of multi-omics data may enable the identification of patient-specific microbial features in CRC and facilitate more precise intervention design (170, 171). However, these strategies remain at an early stage of development, and their clinical feasibility and long-term safety require further validation.

## 6 Conclusion and future perspectives

The interactions between the gut microbiota and the host innate immune system are highly complex and dynamic, and their imbalance has been closely implicated in the initiation, immune evasion, and tumor microenvironment remodeling of CRC. Existing evidence indicates that microbial structural components, metabolites, virulence factors, and nucleic acid–related signals may participate in multiple layers of local immune regulation by influencing pattern recognition receptor activation, myeloid cell function, NK cell activity, and the tumor immune microenvironment. Conversely, the state of the innate immune system and mucosal barrier integrity may also reciprocally shape the composition, spatial distribution, and metabolic characteristics of the gut microbiota, thereby forming a complex bidirectional and dynamic regulatory network. Across disease-related studies, microbiota–innate immune interactions appear to play roles at different stages of CRC development and progression.

At present, microbiota-based interventions—including FMT, probiotics, prebiotics, engineered microbial systems, and bacteriophage therapy—have shown promising potential in modulating gut microbial composition and influencing host immune responses; however, these strategies remain largely exploratory and are still associated with important limitations. Future studies integrating metagenomics, metabolomics, single-cell sequencing, and spatial multi-omics technologies are needed to further elucidate key microbiota–innate immune interaction nodes and functional networks, thereby providing a more robust theoretical foundation and potential therapeutic targets for precision microbiota-based interventions in CRC.
